# Can audience involvement stimulate visit intention in Chinese Kung Fu? Using a serial multiple mediation model to explore film tourism

**DOI:** 10.3389/fpsyg.2022.979839

**Published:** 2022-10-06

**Authors:** Jiangtao Xia, Qihang Qiu, Yifan Zuo, Liang Wang

**Affiliations:** ^1^School of Leisure Sports and Management, Guangzhou Sport University, Guangzhou, China; ^2^Faculty of Human Geography and Planning, Adam Mickiewicz University, Poznań, Poland; ^3^School of Physical Education, Shenzhen University, Shenzhen, China; ^4^School of Physical Education, Yichun University, Yichun, China

**Keywords:** film tourism, Kung Fu, audience involvement, visit intention, destination image, anticipated experience

## Abstract

The popularity of film and television causes audiences to flock to the location of filming, and thus film tourism is widely been used as an efficient tool in destination branding and marketing. Accordingly, this study takes Chinese Kung Fu film and television as an example, using the method of regression model to study audience involvement, cognitive destination image, tourists’ anticipated experiences, and visit intention into an overall framework. Findings illustrate a significant positive correlation among these four factors. Specifically, cognitive destination image and tourists’ anticipated experiences have a partial mediating effect on the relationship between audience involvement and visit intention. This study provides three suggestions for the development of film tourism, namely maximizing the marketing effect, strengthening the unity of film and images of destinations, and making full use of modern marketing tools.

## Introduction

Benefiting from the development of digital technology and capital market, the global film industry is growing rapidly. In recent years, film tourism is widely recognized as a driver of tourism development for many destinations and has become increasingly important as a marketing tool for tourist destinations (Connell, 2012; Wen et al., 2018). One well-known example is that the filming of Game of Thrones has had a profound effect on boosting tourism numbers in Northern Ireland and in Croatia, as well as the country’s image in global awareness (Tkalec et al., 2017; Mannheimer et al., 2022). Similarly, 6% of international tourists flock to New Zealand to see the Lord of the Rings attractions (Roesch, 2009). Besides, according to the Global Tourism Destination Analysis Report released by the China Tourism Research Institute and the Mafengwo Company, 30% of Chinese tourists are influenced by film, television, and reality shows (China Tourism Academy and Mafengwo, 2017).

Previous studies have proved the potential of film in creating an attractive destination image and enhancing tourists’ visit intention. Nevertheless, the development of film and television tourism still depends on the effective combination of film and television with destination tourism resources to continuously attract tourists. Otherwise, film and television tourism will face the risk of “a short life cycle” or “few tourists.” In film and television tourism, audiences change their identity to become tourists, and the degree of audience involvement in a film is often regarded as one of the most crucial factors affecting the development of film and television tourism. With audiences’ deep involvement, film-watching activities can be transformed into an unforgettable and pleasant journey. The more emotional the involvement of the audience in watching series, the more likely they are to visit film and television tourism destinations (Fu et al., 2016). Moreover, the cognitive destination image is another factor affecting tourists’ intentions. A destination image with a positive and visual aspect is likely to be considered and selected during tourists’ travel decision-making process (Pearce, 1982). In addition, tourists’ anticipated experience, a type of tourism expectation developed by audiences through film and television works before visiting a tourist destination (Kim, 2012a), plays an important role in tourists’ visit intentions. Tourists’ anticipated experiences.

Although few studies combine film and television media, cultural communication, and tourism motives from an interdisciplinary perspective (Connell, 2012), research on film and television tourism must still clarify whether audience involvement affects cognitive destination image and tourists’ anticipated experiences, thereby affecting tourists’ destination visit intention. Therefore, this study relies on the theoretical basis of related research on audience involvement to expand the development mechanism and theoretical boundaries of existing theories. This study takes Chinese Kung Fu film and television tourism as a case study, considering its distribution, cultural root, and popularity in China (Zuo et al., 2021). In addition, this study can provide practical strategies for the product development and investment plan of film and television tourism destinations to enhance existing cognition and present suggestions for the development of Kung Fu film tourism from the aspects of the cultural integration of shooting projects, the image construction of shooting locations, and the formation of tourists’ anticipated experiences.

## Literature review and hypotheses

Film tourism refers to shooting the destination as the plot and story background in the video to deepen or change the audience’s impression of the destination, and thus increase tourist number and local economic benefits (Kim et al., 2007). In this study, audience involvement, cognitive destination image, anticipated experiences, and visit intention interact in film tourism.

### Audience involvement

The concept of audience dynamic or active viewing experiences was recognized in media communication research, that is, when choosing and interpreting media input, the audience is an active individual. This discourse regarded the audience as the market, with each consumer having certain needs and preferences for certain content (Taheri et al., 2014). The degree of audience involvement is the main concern of business operators or media producers. Therefore, the theory of “Use and Satisfaction” emerged, which states that audiences are active and are either interactive rather than media-oriented; it also assumes that audiences use media to meet their individual needs. Destination marketers must understand the needs of different audiences to ensure the sustainable development of a destination (Schofield et al., 2020). The higher the degree of audience involvement, the more positively the audience will think or give feedback, connect the media content with their own reality, and discuss the communication content with others (Rauniar et al., 2013). When a similar communication content situation or environment is encountered, audiences will recall the communication content and replace it with their own emotions (Huang, 2013). By contrast, low-involvement audiences are absent-minded and lack the understanding and memory of the communication content (Mcquail, 1997). In addition, the Use and Satisfaction Theory emphasizes that tourism practitioners should recognize the standpoint of the audience through the analysis of the audience’s use of media and the satisfaction of their needs, to examine the psychological and behavioral effects of media on people (Zuo et al., 2022). Second, the result of audience participation often implies whether the needs are met or not met. For film and television tourism, the demand of the audience is often a longing for the tourism destination and an expectation of a tourism experience. Assuming that a tourism destination can meet the needs of the audience, then, they can also effectively form visit intention.

Early studies regarded audience involvement as a parasocial interaction; the latter is considered the simplification of cognitive and emotional responses, interpersonal involvement, emotional bonds, and an important part of audience involvement (Sood and Rogers, 2000). Previous studies also proposed similar concepts and methods, such as indirect involvement through vicarious participation (Riley et al., 1998), empathic involvement (Kim and Richardson, 2003), celebrity involvement (Yen and Croy, 2016), and emotional involvement (Kim and Kim, 2018). Studies found that audiences can increase their degree of involvement by collecting information about a film and buying products related to the film’s content or actors and characters (Sood and Rogers, 2000). In film and television tourism, emotional expression, personal involvement, and the symbolic significance of the film output (especially stories and characters) are emphasized (Kim and Richardson, 2003). For example, among tourists inspired by Korean dramas, their main reason for visiting South Korea is to see the sceneries and buildings featured in such shows (Kim et al., 2007). In the long process of watching a film, audiences may experience a sense of intimacy with the content of the film. Audiences may be completely immersed in the movie situations, react to the actors, and integrate themselves into the film environment (Valaskivi, 2000). These factors are concluded by Kim (2012a) as measurement scales of audience involvement, namely behavioral involvement, emotional involvement, and referential reflection.

In conclusion, audience involvement may affect tourists’ intention to visit a destination (Kim, 2012a). At the same time, previous studies showed that audience involvement can directly or indirectly affect audiences’ intention to visit a filming location (Kim and Kim, 2018), cognitive destination image (Fu et al., 2016), and tourists’ anticipated experiences (Kim, 2012a). Existing studies also demonstrated that movies and television shows are considered the most influential media in building the image of tourist destinations and have a certain impact on the visit intentions of actual and prospective tourists, whose access behavior derives from their cognitive destination image (Sancho and Álvarez, 2010; Muhoho-Minni and Lubbe, 2017). Accordingly, this study makes the following hypotheses:

**Hypothesis 1a:** A positive relationship exists between audience involvement and cognitive destination image.

**Hypothesis 1b:** A positive relationship exists between audience involvement and tourists’ anticipated experiences.

**Hypothesis 1c:** A positive relationship exists between audience involvement and visit intention.

### Tourists’anticipated experiences and the formation of cognitive destination image

Tourists’ anticipated experiences are the starting point and focus of this study. With the help of advertisements, travel brochures, and books, tourism practitioners plan the original ordinary places into tourist consumption destinations full of stories and then market these places that have been narrated (Chronis, 2012) to construct the anticipated experiences of potential tourists. As Urbain (1989) stated, the basic narrative structure has the power of “narrative programming” and the ability to promote a place into a tourism imagination in advertising information. Through a series of media representations, such as books, movies, and television, among others, the commercial development of the narrative place will be further consolidated. Therefore, when marketing a tourist destination, they not only sell a place but more importantly sell the story, meaning, and value of the destination.

Several studies suggested that an internal relationship may exist between audiences’ viewing experiences in a cinema and tourists’ experiences. As a kind of sensory marketing, a movie visual scene can influence individuals’ destination emotion and anticipated experience (Ghosh and Sarkar, 2016). Tourists’ anticipated experiences are a type of tourism expectation developed by audiences through film and television works before visiting a travel destination (Teng and Chen, 2020). Moreover, they are a kind of virtual reality after the consumption of a movie scene and environment interaction (Buchmann et al., 2010). The audience combines their own knowledge structure and new information to generate mind’s vision (Cowan and Ketron, 2019), as if they are interacting with the destination. These interactions stimulate tourists to imagine the destination, form a cognitive destination image and emotional connection, and then affect the anticipated experience. Le et al. (2019) proposed that the contribution of tourists’ cognitive image to the tourists’ anticipated experiences will be the research direction in future.

A tourist destination image is the cognitive or impression of tourists of destinations, which is related to the expected benefit and consumption value (Muhoho-Minni and Lubbe, 2017). A tourism destination image is a comprehensive impression formed by tourists’ cognition, emotions, views, and perspective of a destination (Valaskivi, 2000). Based on substantial literature and a summary of previous theories, several scholars proposed the novel “cognitive-emotional” model of tourism destination image, which divides tourists’ overall image into a cognitive image and an emotional image. Most studies on the topic of tourist destination image emphasize its impact on tourism choices before traveling (Özdemir and Adan, 2014). Tourists’ cognitive image of a certain destination terrain is largely based on images before and during a visit. Affected by film and television dissemination, this type of cognitive destination image is highly specific and better “packaged” and thus can have a positive impact on future visit intention (Prayag, 2009).

From the perspective of tourism destination marketing, tourists’ experiences are directly related to the cognitive destination image. Through film and television exposure, tourists’ anticipated experiences can be promoted and a destination image can be deepened (Connell and Meyer, 2009). Celebrity involvement can affect tourists’ cognitive destination image, thereby enhancing their intention to visit destinations (Lee S. et al., 2015). Chen (2018) found that cognitive destination image has a mediating effect on the relationship between celebrity involvement and place attachment. Tourists’ cognitive destination image reasonably explains how celebrity involvement affects place attachment. In addition, landscape images are the most likely elements to increase visit intention. Hypotheses are therefore as follows:

**Hypothesis 2a:** A positive relationship exists between cognitive destination image and tourists’ anticipated experiences.

**Hypothesis 2b:** A positive relationship exists between cognitive destination image and visit intention.

### The formation of visit intention

**FIGURE 1:**
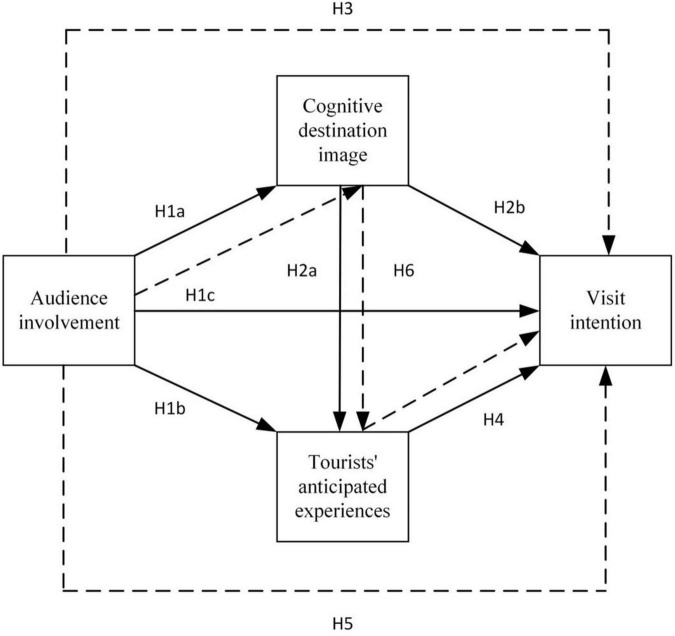
Conceptual model.

Although film and television do not always induce tourism, it has the potential to increase visit intention to the destination through a comprehensive system with audience involvement, cognitive destination, and anticipated experience. Previous studies showed that audience involvement or degree of involvement directly affects audiences’ live film and television tourism experiences at original filming locations and audiences’ behaviors are driven by their tourists’ anticipated experiences (Kim, 2012a). In Kim’s (2012b) study, the results show that tourists’ anticipated and on-site experiences have a significant impact on satisfaction, visit intention, revisit intention, and recommendation intention. The assumption that the tourists’ anticipated experiences will affect their visit intention is put forward. Furthermore, cognitive destination image and potential tourist experiences have a chain-like mediating effect on the relationship between audience involvement and visit intention. In other words, the chain mediating effect of cognitive destination image and tourists’ anticipated experiences is more obvious than the single mediating effect of tourists’ anticipated experiences. Hypotheses are, therefore, as follows:

**Hypothesis 3:** Cognitive destination image has a mediating effect on the relationship between audience involvement and visit intention. Figure 1 illustrates the conceptual model of the mediation effect.

**Hypothesis 4:** A positive relationship exists between tourists’ anticipated experiences and visit intention.

**Hypothesis 5:** Tourists’ anticipated experiences have a mediating effect on the relationship between audience involvement and visit intention.

**Hypothesis 6:** Cognitive destination image and tourists’ anticipated experiences have chain-like mediating effects on the relationship between audience involvement and visit intention.

Figure 1 illustrates the conceptual model of the mediation effect.

## Research method

### Case survey

This study selected Foshan, a tourist city in southern China with a long history and become famous because of Kung Fu, as case study. Since the last century, *Huang Fei Hong* series (since 1949), *Ip Man* series (2008–2019), *Bruce Lee* series (1971–1973), *Cai Lifo* (2011), and other film and television works made Chinese Kung Fu famous worldwide. Especially, *Ip Man* series, starring Chinese actor Donnie Yen, achieved box office and word-of-mouth success and opened up a new vision of martial arts films in Asia. As the hometown of Ye Wen and Huang Fei Hong, Foshan has become nearly synonymous with modern Chinese Kung Fu movies. The Huang Feihong Memorial Hall and Yewen Hall, which are located in an ancestral temple, are the most famous scenic spots in Foshan. In Chinese Kung Fu films and television shows, many scenic resources are from the Foshan area, such as Xiqiao Mountain, the Foshan Ancestral Temple, the Nanfeng Ancient Kiln, Qinghui Garden, Nanguo Peach Garden, and other cultural and historical landscapes as well as rich subtropical natural landscapes (Heikkila, 2011).

### Measurement instrument

Based on previous experiences, this study developed a questionnaire survey in accordance with standardized procedures (Ouyang et al., 2017). Given that the survey was conducted in China, the questionnaire was translated into Chinese according to the reverse translation procedure (Brislin, 1970). Three scholars and two research assistants evaluated the content validity of the items in the scale used to measure each construct; they also assessed the content and comprehensibility of the measurement items. Moreover, they identified items that should be re-edited and improved to enhance clarity, readability, and content effectiveness. These five researchers also determined whether the items were redundant and recommended the improvement of the quota items of each construct. To test the feasibility and reliability of the tool, a pilot test was first carried out from May 2 to 5, 2020 on a group of 50 social media users, between the ages of 18 and 50 years old, in China. The pre-survey aims to attempt to improve the quality of questionnaire, delete the unclear items, refine the survey content and structure, and preliminarily verify the reliability and validity of the scale.

This study adopted the Audience Involvement Scale developed by Kim (2012a). The questionnaire included 15 questions, which were divided into three dimensions. In this study, the total internal consistency coefficient of the questionnaire is 0.925, and the internal consistency coefficients of the three dimensions, that is, behavioral involvement, emotional involvement, and referential reflection, are 0.835, 0.859, and 0.821, respectively. The revised questionnaire demonstrated satisfactory structural validity, and the fitting indices of the confirmatory factor analysis are χ^2^/*df* = 2.734, RMSEA = 0.072, GFI = 0.910, NFI = 0.912, IFI = 0.943, TLI = 0.928, and CFI = 0.942.

This study adopted the tourists’ cognitive destination image scale developed by Gearing et al. (1974). The questionnaire included 14 questions, which were divided into three dimensions, that is, quality of experience, attractions, and value/environment. The total internal consistency coefficient of the questionnaire is 0.935, and the internal consistency coefficients of the three dimensions, that is, quality of experience, attractions, and value/environment, are 0.899, 0.840, and 0.790, respectively. The fitting indices of the confirmatory factor analysis are χ^2^/*df* = 2.846, RMSEA = 0.075, GFI = 0.904, NFI = 0.924, IFI = 0.950, TLI = 0.937, and CFI = 0.949.

This study adopted the film and television anticipated experiences scale developed by Kim et al. (2007). The scale is related to sensory/symbolic experiences, experiences beyond the screen, and celebrity- and character-oriented experiences. The questionnaire included 12 questions, which were divided into three dimensions. The total internal consistency coefficient of the questionnaire is 0.938, and the internal consistency coefficients of the three dimensions, that is, prestige and privilege, sensory experiences beyond the screen and reenactment, and intimacy and memory, are 0.875, 0.845, and 0.831, respectively. The fitting indices of the confirmatory factor analysis are χ^2^/*df* = 2.253, RMSEA = 0.062, GFI = 0.945, NFI = 0.955, IFI = 0.974, TLI = 0.966, and CFI = 0.974.

This study adopted the tourist visit intention vector scale developed by Baker and Crompton (2000). The original scale measured tourists’ willingness to visit museums through three items. The questionnaire included three questions, and the total internal consistency coefficient of the questionnaire is 0.815.

### Data collection

The sample population of this study was audiences who have watched Foshan Kung Fu films and television shows but have never visited Foshan. Data collection was conducted from May 30 to June 15, 2020. This study used convenience sampling, which is a non-probability sampling technique, to distribute the questionnaires online and through social software to different communities. Finally, a total of 331 data samples were collected from 33 provinces and autonomous regions in China, except Tibet, including a small number of samples from Ireland, Malaysia, Japan, and Singapore (international students who live in Guangzhou and know Chinese), as shown in Table 1.

**TABLE 1:** Descriptive statistics of demographic characteristics.

Respondents profile (*N* = 331)		

Demographics	Total number	Percentage (%)
**Gender**		
Male	181	54.7
Female	150	45.3
**Age**		
Below 18 years	1	0.3
18–25 years	188	56.8
26–35 years	119	36.0
36–50 years	16	4.8
51–60 years	6	1.8
Above 60 years	1	0.3
**Education**		
High school (technical secondary school) and below	15	4.5
Junior college	29	8.8
Undergraduate	138	41.7
Graduate or above	149	45.0

The data were analyzed using Statistical Package for the Social Sciences (SPSS 22.0). First, descriptive statistics were performed on the demographic characteristics of the respondents. Next, Cronbach’s alpha was used to check the internal consistency of each construct, and the results showes that reliability is acceptable. Subsequently, this study used AMOS 24.0 for confirmatory factor analysis (CFA). CFA was conducted to check the aggregate validity of the questionnaire. Then, the descriptive statistics and correlation analysis results of each variable were counted. To test the main effect of the model, a series of multiple regression analyses were initially performed. Finally, bootstrapping was used to test the mediating effects. Specifically, this study selected Model 6, adopted 5,000 bootstrap samples, and selected the 95% confidence interval.

## Results

### Demographic profile

The results showes that all the variables are significantly correlated. Combined with the mean value of each variable, the results presents that the subjects have a high degree of involvement in film and television works, possess a certain positive cognition of Foshan from the film and television works, achieve improved tourists’ anticipated experiences, and demonstrate high intention to visit. The fitting indices of the final CFA model are improved, that is, χ^2^/*df* = 1.523, RMSEA = 0.040, GFI = 0.857, NFI = 0.879, IFI = 0.955, TLI = 0.948, and CFI = 0.954. Meanwhile, the standardized factor load of each item is greater than 0.5 and less than 0.9, thereby indicating that the aggregation validity is satisfactory (Shown in Table 2).

**TABLE 2:** Descriptive statistics and correlation analysis results of each research variable (*N* = 331).

	M	SD	AI	PC	PTE	VI
Audience involvement	3.78	0.64	1			
Cognitive destination image	4.00	0.56	0.68**	1		
Tourists’ anticipated experiences	4.04	0.63	0.69**	0.81**	1	
Visit intention	4.04	0.72	0.59**	0.70**	0.70**	1

The mean value (M) in the table is the average of all the index scores of each variable, and the standard deviation (SD) is calculated on the basis of the average of each variable index. In the table, AI represents audience involvement, PC represents cognitive destination image, TAE represents tourists’ anticipated experiences, and VI represents visit intention. **p < 0.01.

### Hypothesis testing

#### Main effects

As shown in Table 3, a significant positive correlation exists between audience involvement and visit intention (coefficient = 0.146, *p* < 0.05). After the audience involvement of the viewers is controlled, the results showes that a significant positive correlation existed between cognitive destination image and visit intention (coefficient = 0.444, *p* < 0.05) as well as between tourists’ anticipated experiences and visit intention (coefficient = 0.382, *p* < 0.05). These results support Hypotheses 2c, 2b, and 4.

**TABLE 3:** Regressions analyses.

DV	IVs	B	SE	*t*	*P*	95% Confidence interval		Hypothesis
							
						LLCI	ULCI	
PC	AI	0.595	0.035	16.946	0.000	0.526	0.664	H1a (S)
	*R* ^2^	0.466				*F* = 287.154, *p* < 0.0001	
TAE	PC	0.713	0.047	15.213	0.000	0.621	0.806	H2a (S)
	AI	0.247	0.041	6.043	0.000	0.167	0.327	H1b (S)
	*R* ^2^	0.692	*F* = 368.436, *p* < 0.0001					
VI	PC	0.444	0.086	5.196	0.000	0.276	0.613	H2b (S)
	TAE	0.382	0.077	4.955	0.000	0.230	0.534	H4 (S)
	AI	0.146	0.060	2.423	0.016	0.027	0.264	H2c (S)
	*R* ^2^	0.550	*F* = 133.319, *p* < 0.0001					

To control audience involvement, the relationship between film and television cognitive destination image and tourists’ anticipated experiences is regressed. The results illustrate that cognitive destination image also has a significant positive impact on the formation of tourists’ anticipated experiences (coefficient = 0.713, *p* < 0.05). These results support Hypothesis 2a. The results demonstrate that the higher the audience involvement, the greater the cognitive destination image (coefficient = 0.595, *p* < 0.05), and tourists’ anticipated experiences increase significantly (coefficient = 0.713, *p* < 0.05). These results support Hypotheses 1a, 1b, and 1c.

#### Mediation effects

To test the impact of multiple mediation effects on visit intention, a series of mediation analyses were performed using Hayes’s Model 6 and bootstrap methods. Table 4 shows the results of all the paths (Hayes, 2012). The results illustrate that under the influence of chain mediation, audience involvement has a direct (coefficient = 0.521, 95% CI: [0.408, 0.652]) significant impact on audiences’ visit intention. The indirect impact of audience involvement on audiences’ visit intention is verified by the significant mediators of cognitive destination image (coefficient = 0.265, 95% CI: [0.154, 0.395]) and tourists’ anticipated experiences (coefficient = 0.094, 95% CI: [0.044, 0.170]). A significant impact is exerted by the mediating chain of cognitive destination image and tourists’ anticipated experiences (coefficient = 0.162, 95% CI: [0.088, 0.244]). These results supported Hypotheses 3, 5, and 6.

**TABLE 4:** Regression coefficients of serial mediation models using process.

Model	Effect	SE	95% Confidence interval	
			
			Boot lower	Boot upper
AI → PC → VI	0.265	0.061	0.154	0.395
AI → TAE → VI	0.094	0.031	0.044	0.170
AI → PC → TAE → VI	0.162	0.039	0.088	0.244

## Discussion

### Influence of audience involvement on tourists’ visit intention

First, from the correlation between audience involvement and tourists’ visit intention and the main effect test results, the higher the audience involvement in Chinese Kung Fu films, the higher the possibility of the audience traveling to the filming location and becoming a tourist. This study combines audience participation with tourist behavior, which provides a certain theoretical basis for the integration of communication and tourism research in future. To put it simply, if the audience is highly involved in the film and television content (e.g., characters, stars, narration, plot, music soundtrack, etc.), then, they are more likely to travel to the places shown in the film and television.

Second, this study not only verifies the empirical reliability and validity of audience participation and visit intention model but also confirms the multi-dimensional research on audience participation in the field of communication (Sood, 2002). This study believes that audience involvement has three dimensions: behavioral involvement, emotional involvement, and referential reflection. However, this study draws different conclusions from previous studies (Kim, 2012a). The audience not only locates Chinese Kung Fu films for entertainment and leisure but also places certain significance on national education, which is the combination of entertainment topics and national education. Consequently, Chinese Kung Fu films are recognized by the audience because of their national and cultural characteristics, storyline, and significance to youth education, and tourism, among others. Especially under the influence of factors such as the construction of Foshan Kung Fu City and national self-confidence education, Chinese Kung Fu films can often gain a good reputation, thereby making the tourism effect very obvious. In addition, a special interpersonal relationship exists between the audience and the film and television characters. With the deepening of the plot, the input of the audience, the continuity of the episode, and other factors, this relationship becomes increasingly intimate, which affects the audience’s self-efficacy, collective efficacy, individual behavior, and collective behavior (Kim, 2012a). Therefore, film and television tourism must strengthen the direct contact between a film and the filming location and increase audience involvement by strengthening the interaction efficiency between the filming location and the audience.

### Mediating the role of cognitive destination image and tourists’ anticipated experiences

First, audience involvement in film and television works affects audiences’ overall cognitive destination image. A positive cognitive destination image can trigger audiences’ desire to travel, which can in turn affect their intention to travel to a certain destination (Fu et al., 2016). However, Kim and Richardson (2003) proved that celebrity involvement positively affected familiarity and visitation intentions, while Lee et al. (2008) verified that empathic involvement with the film characters was not significantly associated with either component (cognitive or affective) of destination image or with familiarity, and the movie did not enhance the degree of familiarity with the destination portrayed in it. These two studies on tourism destination image have different results, which cannot prove the positive impact of alternative or celebrity participation on film and television tourism cognitive destination image. Therefore, further research is needed to refine the concept of audience involvement and to better understand the role of audience involvement in cognitive destination image and tourists’ anticipated experiences.

An interactive relationship exists between the audience and the film rather than the media leading the audience. However, only when tourism destination marketing organizations understand the needs of different audiences through film and television works can they achieve the sustainable development of film and television tourism destinations (Schofield et al., 2020). As mentioned in the Use and Satisfaction Theory, the more audiences participate, the more positive they think or give feedback. The audience connects the media content with their own reality, and assumes that they enter the environment where the communication content is similar, recalls the communication content, substitutes it with their own emotions, and finally forms the behavior intention to meet the psychological needs (Mcquail, 1997). This study effectively confirms that the audience’s cognition of the destination image is to recall the relevant scenes in the film and television drama, and the pre-travel experience is the result of mixing their own emotions, which will eventually form a visit intention. The smooth formation of film and television tourism visit intention depends on the high-quality integration of filming location image and tourists’ anticipated experiences (Rauniar et al., 2013).

In addition, the degree of audience involvement has a significant positive impact on cognitive destination image. A film can shape the image of the filming location and present it to audiences (Chen, 2018). In the study of film and television tourism in the Dachangjin Theme Park (Kim, 2012b) and Nami Island, where “Winter Sonata” was filmed (Kim et al., 2007), the audience’s emotional participation not only can predict their emotional memory and ownership in the tourist destination but also create a special tourism space. This kind of space exists only because of tourists’ emotional identity and empathy for film and television works. Moreover, the degree of audience involvement has a significant positive impact on tourists’ anticipated experiences. That is, when audiences experience a high degree of audience involvement in the television content (e.g., characters and celebrities, narratives and storylines, and music), their anticipated experiences in the filming locations will become highly positive (Kim, 2012a).

The multi-chain mediation path based on cognitive destination image-potential tourist experiences is better than the single intermediary variable mediation path of potential tourist experiences. This finding shows that the mechanism of potential tourist experiences in audience involvement in audiences’ visit intention must be performed by optimizing cognitive destination image. When the audience perceives the image of the tourism destination, this symbolic meaning and value are immediately reflected in the specific film and television tourism space, attraction, and tourists’ pre-experience. In the stage of anticipated experiences, tourists construct their imagination and anticipated experiences of the destination with the help of film and television works. Therefore, the audience’s recognition and empathy of the program can predict their cognitive destination image of film and television destinations and pre-travel experience, which is also the root cause of film and television tourism phenomenon and behavior. In a word, the influence of film and television works on audience visit intention is not the result of works alone, among which the influencing factors involve the content, audience involvement, and tourism destination, which together constitute the influence process mechanism of film tourism.

## Conclusion and implications

### Conclusion

This study aims to explore how films arouse interest in certain destinations, and how to attract tourists and form tourist visit intention. The results show that audience involvement is positively correlated with tourists’ visit intention; whereas cognitive destination image and tourists’ anticipated experiences play a mediating role. In addition, the multi-chain mediating path of cognitive destination image-tourists’ anticipated experiences is better than the single intermediary variable mediating path of tourists’ anticipated experiences. The empirical results support the theoretical model and hypotheses.

### Implications

First, this research together with previous studies have found that film and television can significantly influence, change, or strengthen the destination image and stimulate the audience’s interest in visiting. With the increasingly fierce competition between international and domestic tourist destinations, the influence of traditional advertising and promotion methods is slowly weakening. Tourism destination marketing organizations and other relevant departments are increasingly aware that film and television play an important role in shaping tourism image, effectively positioning, stimulating tourism demand, and increasing the number of tourists, among others. Local governments no longer passively accept the various effects of the broadcast of film and television on the local tourism industry but actively exploit and fully tap the marketing opportunities brought about by the broadcasting of films and television. In the shooting of film and television, the local authorities should maintain good public relations with the media, mobilize the media to publicize and report the film and television and their filming places, maintain a high exposure rate, and invite travel agents to study tours or attend special press conferences. After the release of film and television, the organizer should actively carry out a series of promotional activities, such as developing film and television tourism products, making film and television maps, and promoting reception facilities such as restaurants and restaurants that appear in film and television. The local government can also set up a special website for potential tourists and conduct joint promotions with tour operators or related organizations. In addition, the potential of film and television tourism can be fully tapped to maximize the marketing effect of film and television tourism. For example, the local government can invite the main actors and actresses to act as ambassadors of the destination image; maintain or copy the iconic symbols, scenes, and facilities in the film and television play; sell film and television memorabilia; and hold film and television festivals (Hudson and Ritchie, 2006).

Second, the image presented in a film and audiences’ anticipated experiences must meet the reality. In the process of visiting the destination, tourists not only experience the objective attributes of the destination but also practice their imagination. Therefore, a destination should design the on-site tourism experience on the basis of the full understanding of the tourists’ expected experience and narrow the gap between the screen visual scene and the on-site tourism experience. By strengthening the unity of film and television works and the images of destinations, tourists’ visit intention induced by a film can be increased. If the film and television tourism destination are more based on natural scenery and rare landscapes, the stakeholder can design and build theme entertainment facilities and create a combination of performance and interaction with the help of the story scenes and romantic events displayed in the film and television. If it is a tourist destination with strong national characteristics and cultural heritage, cultural experience tourism can be developed to enhance the participation of tourists.

Third, tourism destination management organizations should also make full use of modern marketing tools such as the Internet and social media. The destination shares information related to film and television shooting through the official website, social media; and interacts with users, such as award-winning retweets, and comments, to name a few. Apart from these virtual marketing tools, brand ambassadors are also a marketing “tool.” In previous studies, many tourism destinations invited celebrity as their ambassadors, making use of their star effect to expand their influence. The celebrity effect stimulates other audiences to know more about tourist destinations (Lee B. et al., 2015; Teng and Chen, 2020). In this era of vigorous development of film and television tourism, there are vivid examples of film and television promoting tourism. For instance, The Foshan government has signed agreements with some film companies and plans to build Foshan into a film city integrating film and television production, technology research and development, copyright trading, achievement exhibition, talent training, and film and television tourism.

### Limitations and future research

Although this study provides valuable management insights for the sustainable development of tourist destinations, several limitations exist. One of them is that cross-cultural research was not conducted on the sample because the research object involves cultural elements. Thus, future research can explore cross-cultural aspects for international tourists or cross-cultural tourists. In addition, common problems related to the measurement errors of dependent and independent variables exist in linear models. Finally, the current study adopts a cross-sectional approach, and the data are sent to the respondents at a specific time through a questionnaire. In view of audience participation in the follow-up process of watching films, audiences’ intention to visit can be affected over a long period, and performance results may be observed in the long run. Therefore, this study suggests that a longitudinal study design be used in future studies to better illustrate causality.

## Data availability statement

The raw data supporting the conclusions of this manuscript will be made available by the authors to any qualified researcher. Requests to access the datasets should be directed to the corresponding author.

## Author contributions

JX: conceptualization, formal analysis, and writing—original draft. JX and YZ: funding acquisition. JX, QQ, LW, and YZ: writing—review and editing. All authors have read and agreed to the published version of the manuscript.
